# E-cigarette use, perceived risks, attitudes, opinions of e-cigarette policies, and associated factors among Thai university students

**DOI:** 10.18332/tid/186536

**Published:** 2024-05-11

**Authors:** Tippanart Vichayanrat, Warungkana Chidchuangchai, Raksanan Karawekpanyawong, Kanisorn Phienudomkitlert, Nichawee Chongcharoenjai, Naphada Fungkiat

**Affiliations:** 1 Department of Community Dentistry, Faculty of Dentistry, Mahidol University, Bangkok, Thailand; 2 Department of Oral Medicine and Periodontology, Faculty of Dentistry, Mahidol University, Bangkok, Thailand; 3 Faculty of Dentistry, Mahidol University, Bangkok, Thailand

**Keywords:** e-cigarettes, university students, health, perceived risk, policy

## Abstract

**INTRODUCTION:**

Although many countries, including Thailand, currently ban the sale of e-cigarettes, their use continues to rise, especially among young adults. Since the study of e-cigarette use among university students is limited, this study aimed to determine factors associated with e-cigarette use and explore university students’ attitudes toward e-cigarettes, perceived risk, and opinion of e-cigarette policies.

**METHODS:**

This cross-sectional study was conducted among undergraduate students using convenience sampling in a university, in central Thailand from November 2022 to February 2023. A self-administered online questionnaire was distributed to 19 faculties representing health sciences, science and technology, social and arts faculties, and the International College.

**RESULTS:**

A total of 548 students completed the online questionnaire, and 20.4% reported ever using e-cigarettes, while 40% of e-cigarette users were unsure about the nicotine content. About 28% agreed, and 22% were unsure whether e-cigarettes could help quit smoking. Most students perceived that e-cigarettes are addictive and harmful, while about half of the participants agreed with the policy related to e-cigarettes in Thailand. Students with positive attitudes towards e-cigarettes were more likely to use e-cigarettes (AOR=1.15; 95% CI: 1.08–1.22), and those with lower perceived risk (AOR=0.89; 95% CI: 0.82–0.96) and who disagreed with e-cigarettes policy (AOR=0.93; 95% CI: 0.89–0.97) were more likely to use e-cigarettes. Personal income and having friends who use e-cigarettes were the significant predictors for e-cigarette use, while studying in the faculty of science and technology was a predictor of using e-cigarettes last month.

**CONCLUSIONS:**

Although the perceived risk was high, about half of the students thought that e-cigarettes could help them quit smoking and were unsure or disagreed with e-cigarette banning policies. Attitude, perceived risk, policy opinions, personal income, and having friends who used e-cigarettes, were associated with e-cigarette use. Thus, correcting misunderstandings and increasing risk perceptions about e-cigarettes must be advocated among university students.

## INTRODUCTION

Electronic nicotine delivery systems (ENDS) are battery-run cartridge devices that generate heat to vaporize electronic liquid (e-liquid) inside the cartridge to create an aerosol the user inhales^1^. E-liquid contains nicotine, flavored compounds, propylene glycol, glycerin, and other substances that could pose health hazards to individuals^2,3^. Alternative electronic non-nicotine delivery systems (ENNDS) exist, which are similar to ENDS yet typically do not contain nicotine in the liquid^1^. The most common ENDS are electronic cigarettes (e-cigarettes), which vary in shape, size, and type, known by numerous names like vapes, vape pens, mods, pod-mods, tanks, dab pens, dab rings, and are continually evolving in design^4^. Research suggests that e-cigarettes are linked to various systemic health conditions^5^, including diminished attention control, impaired learning ability, altered mood, and increased impulsivity^6^. Additionally, their use has been associated with incidents of e-cigarette or vaping product use-associated lung injury (EVALI)^7,8^. Moreover, previous studies have highlighted the risks of secondhand vapor exposure^9^ and thirdhand vapor exposure from e-cigarettes^10^. In terms of oral health, e-cigarette use is associated with an elevated risk of dental caries, toothache, and periodontal disease^11,12^. Sweet-flavored e-liquids and chemicals may further increase the likelihood of cariogenic potential^13^. Furthermore, e-cigarette users are more prone to experiencing oral lacerations, teeth fractures, and avulsions resulting from e-cigarette explosions^12^.

The current prevalence of e-cigarettes among university students has risen continuously as they are perceived to be better than cigarettes and less harmful^14^. The rise is also linked to the absence of cigarette smoke odor, the availability of flavors, self-curiosity, alluring advertisements, smoking cessation, and peer and family use^15,16^. A national survey in the US among adults and young adults revealed that about half of the participants believed that e-cigarettes were less harmful than cigarettes and associated with the past 30-day e-cigarette use^17^. A previous study in Jordan indicated that 11% of university students reported e-cigarette use, and 26.5% of e-cigarette users reported using it for smoking cessation^18^. A recent study reported that 13.5% of medical students in Slovakia currently use e-cigarettes^19^. A study in Hanoi showed that 13.2% of university students used e-cigarettes^20^. In 2020, 18.1% of university students in northern Thailand reported currently using e-cigarettes, with the majority of users switching between e-cigarettes and conventional cigarettes^21^.

Many countries have different policies about e-cigarettes. The responses to e-cigarette policies across Southeast Asian countries differ significantly, from stringent bans to limited or absent regulations^22^. Thailand is one of five countries in the Southeast Asian region that has banned e-cigarettes and vaping products. However, there remains a lack of knowledge about the perceived risk of and attitudes towards e-cigarette use, which could be associated with health risk behavior and policy opinions about using e-cigarettes in university students. Since data on e-cigarette use among university students in Thailand are scarce, this study aimed to identify the factors associated with e-cigarette experiences, attitudes, risk perceptions, and opinions of e-cigarette laws and policies in Thailand.

## METHODS

### Study design and participants

This cross-sectional study was conducted among undergraduate students in a university in central Thailand from November 2022 to February 2023. The inclusion criteria included being aged ≥18 years, being able to access online questionnaires and reading and writing the Thai language. All 19 faculties were divided into four groups representing: Health Science, Science and Technology, Social Sciences and Arts, and the International College. The proportions of students in these four groups were calculated to represent the proportions of students in the associated university faculties. The researchers randomly selected participants from each university faculty until each group’s required number of students was reached.

### Sample size calculation

We calculated the sample size using the formula^23^:


$$\text{n}=\frac{Z^{2}p\left(1-p\right)}{d^{2}}$$

In a previous study, the prevalence of students who used e-cigarettes was 7.6% (p=0.076)^20^. The confidence level was 95% (the standard value of 1.96; Z), and the margin of error was 3% (d=0.03). The required sample was at least 296. Given the expected response rate of 50%, the required sample size was at least 600. The sampling frames for the Health Science, Science and Technology, Social Sciences and Arts, and International College were 60%, 25%, 7.5%, and 7.5%, respectively.

### Questionnaire development

The self-administered online questionnaire was developed using the Google Forms platform and modified from the previous study^24^. Based on a literature review, the researchers’ discussions, and pilot testing, the questions regarding perceived risk and policy opinions were added, while the attitude items were adjusted and reduced to reach internal consistency reliability.

The questionnaire contained both closed- and open-ended questions and included the following six main sections: demographic information, use of e-cigarettes, e-cigarettes and media, attitudes, risk perception of e-cigarette use, and opinions regarding e-cigarette policies. The demographic information section consisted of seven questions on age, gender, years of study, faculty, accumulated grade point average (GPA), personal monthly income, and family income. The use of e-cigarettes section consisted of nine questions: two questions required a short numeric answer, and the rest used a checklist.

### Measurement

Current e-cigarette use was measured using the question: ‘During the past 30 days, did you use any e-cigarettes?’. Responses were classified as yes and no. Use of e-cigarettes at least once was identified if the respondent answered ‘yes’ to the question: ‘Have you ever used e-cigarettes?’. Perceived risk (4 items), attitudes toward e-cigarettes (5 items), and policy opinions (5 items) were measured on a 5-point Likert scale (1=totally disagree, 2=disagree, 3=unsure, 4=agree, 5=totally agree). Perceived risks of e-cigarettes were defined as perceptions of addiction, harmful to the lungs and cardiovascular system, causing oral health problems, and the secondhand exposure. Attitudes toward e-cigarettes were defined as the agreement that e-cigarettes could help quit smoking, are safer than traditional cigarettes, help to get along with friends, and have trendy images and looks to make them want to try. Policy opinions were based on Thai laws and regulations, which deem e-cigarettes illegal, prohibit their importation, stipulate penalties including imprisonment and fines, restrict online sales, and prohibit possession.

### Validity and reliability of the questionnaire

The content validity index (CVI) assessed the questionnaire’s validity. Three experts in behavioral science and tobacco use were asked to rate the relevancy of each question to the objective of the question. An item content validity index (I-CVI) of more than 0.7 was acceptable^25^. A pilot study of 30 students from other universities was used to determine the questionnaire’s reliability, which was calculated using Cronbach’s alpha. The questions in the perceived risk and attitude scales were revised and adjusted until Cronbach’s alpha was more than 0.7, which is considered acceptable^26^.

### Data collection

The students from each faculty were tabulated and divided into four groups: Health Science, Science and Technology, Social Sciences and Arts, and International College. The estimated number of faculty members was then identified based on the ratio of students required in these four groups. Medicine, Dentistry, Pharmacy, and Public Health faculties were selected for the Health Science group. The faculties of Engineering, and Science, were selected for the Science and Technology group. The faculties of Liberal Arts, and Environment and Resource Studies, were selected for the Social Sciences and Arts group. The International College represented the International College group. Finally, the investigators approached the faculty representatives, mainly student union presidents, to begin the participant recruitment. Participants were invited to participate in the questionnaire via an infographic to advertise the project, distributed online and on-site. Where the on-site year group representatives could be contacted, the investigator visited the students on campus to enhance the recruitment process and increase the number of participants in the study.

Information and consent forms were distributed to all students online. Questionnaire completion reminders were sent to increase the response rate. These were set at three intervals: 1 week, 2 weeks, and one month after the initial distribution of the questionnaire.

### Data analysis

The statistical analyses were conducted using SPSS software. Descriptive analysis, including calculation of frequencies, percentages, means and standard deviations, was used to describe the student’s characteristics, the prevalence of e-cigarette use, attitudes, and risk perception about e-cigarette use. The dependent variables were current e-cigarette use behavior and used e-cigarettes at least once. The independent variables were student characteristics (i.e. age, gender, grade, faculty, family and personal monthly income, and having friends or family who use e-cigarettes), attitudes towards e-cigarette use, risk perceptions of e-cigarette use, and opinions on policies about e-cigarette use in Thailand. Chi-squared, Mann-Whitney U tests, and logistic regression were used to determine the relationships between the independent and dependent variables. The statistical significance level was set at 0.05; all tests were two-tailed.

## RESULTS

### Participant characteristics and e-cigarette use

Table 1 shows the characteristics and e-cigarette experiences of the study participants. Among 548 participants who completed the online questionnaire, the majority were female (71.5%), and the average age was 20.5 ± 1.5 years. Most participants were from the health sciences faculties (66.4%) and science and technology faculties (15.5%). Around half of the participants were in the second and third years of study, and around half had a monthly personal income of 5001–10000 THB (1000 Thai Baht about US$27). Three-quarters of the participants reported having a GPA of 3.01–4.00.

**Table 1:** Characteristics and e-cigarette experiences among Thai university students, 2022–2023 (N=548)

*Characteristics*	*n*	*%*
**Gender** (Female)	392	71.5
**Age** (years)		
18–20	282	51.4
21–23	247	45.1
24–26	19	3.5
Mean (SD), Range	20.5 (1.5), 18–26	
**Faculties**		
Health Sciences	364	66.4
Science and Technology	85	15.5
Social and Arts	63	11.5
International College	36	6.6
**GPA**		
<2.00	15	2.7
2.01–3.00	122	22.3
3.01–4.00	411	75.0
**Family monthly income** (THB)		
≤30000	140	25.5
30001–50000	127	23.2
50001–100000	149	27.2
>100000	132	24.1
**Personal monthly income** (THB)		
≤ 5000	145	26.4
5001–10000	276	50.4
>10000	127	23.2
**Ever use e-cigarettes**	112	20.4
**Type of e-cigarettes used**		
Contain nicotine	59	52.7
No nicotine	8	7.1
Unknown/not sure	45	40.2
**Age started using e-cigarettes** (years)		
14–16	14	12.5
17–18	31	27.7
19–20	36	32.1
>20	12	10.7
Missing data	19	17.0
**Used e-cigarettes during last month** (N=112)	49	43.8
**Source of e-cigarettes last month** (N=49)		
Did not buy	24	49.0
Online	11	22.4
Others (i.e. friends)	3	6.1
Shops	5	10.2
No response	6	12.2
**Think of quitting e-cigarettes** (N=112)	75	81.5
**Any family member uses e-cigarettes** (N=548)	76	13.9
**Any friends use e-cigarettes** (N=548)	407	74.3

THB: 1000 Thai Baht about US$27.

Around 20% of the participants reported ever using e-cigarettes, which mostly contained nicotine, while about 40% of them were unsure about the nicotine content. Most of the participants began using e-cigarettes when they were aged 17–20 years. Among those who reported ever using e-cigarettes (112 participants), around 44% had used them during the last month. On average, participants who used e-cigarettes during the last month used them for 1 to 10 days, and 1 to 10 times per day. However, 26.5% (13 participants) reported using e-cigarettes for more than 20 days in the previous month, and 14.3% used them >20 times per day. Around 74% reported having friends who used e-cigarettes, while 14% had family members who used e-cigarettes. Most participants who bought e-cigarettes reported buying them online. Most of those who had ever used e-cigarettes said they were thinking of quitting (81.5%).

The main reasons participants started using e-cigarettes were wanting to try them (33%) and because a friend used them (31.1%). The most frequent times that e-cigarettes were used were at parties (72.3%) and when drinking alcohol (61.6%). Most participants saw e-cigarettes in the media less than once a month. This was mainly on Instagram (51.8%), Facebook (48.4%), and TikTok (43.5%) (Supplementary file Figures 1–3).

### Perceived risk, attitudes towards e-cigarette use, and policy opinion

Table 2 shows the participants’ perceived risk, attitudes, and policy opinions. Most of the participants agreed that e-cigarettes could harm the lungs and cardiovascular system (90.7%), that e-cigarettes were addictive (88.9%), could cause oral health problems (83.4%), and that secondhand exposure to e-cigarette vapor was harmful (81.9%).

**Table 2:** Perceived risk, attitudes toward e-cigarettes, and policy opinions among Thai university students (N=548)

*Characteristics*	*n (%)*			*Score*
	*Totally disagree/disagree*	*Not sure*	*Agree/ totally agree*	*Mean (SD)*
**Perceived risk of e-cigarettes**				
Addictive	21 (3.8)	40 (7.3)	487 (88.9)	4.5 (0.9)
Can harm the lungs and cardiovascular system	18 (3.3)	33 (6.0)	497 (90.7)	4.6 (0.8)
Can cause oral health problems	37 (6.8)	54 (9.9)	457 (83.4)	4.4 (1.0)
Secondhand vapor exposure is harmful	45 (8.2)	54 (9.9)	449 (81.9)	**4.3 (1.1)**
**Attitudes towards e-cigarettes**				
Could help to quit smoking	270 (49.3)	120 (21.9)	158 (28.8)	**2.5 (1.4)**
Safer than traditional cigarettes	398 (72.6)	76 (13.9)	74 (13.5)	1.9 (1.2)
Help to get along with friends	425 (77.6)	61 (11.1)	62 (11.3)	1.8 (1.1)
Using them shows that you are the new generation and trendy	433 (79.0)	55 (10.0)	60 (11.0)	1.7 (1.1)
The look and image of e-cigarettes make me want to try	408 (74.5)	58 (10.5)	82 (15.0)	1.8 (1.3)
**Policy opinions on e-cigarettes**				
Are illegal	142 (25.9)	124 (22.6)	282 (51.5)	3.5 (1.4)
Importation is strictly prohibited	164 (29.9)	129 (23.5)	255 (46.5)	**3.3 (1.4)**
Anyone who imports them is sentenced to jail for up to 10 years and/or fined 5 times the cost of e-cigarettes	153 (28.0)	118 (21.5)	277 (50.5)	3.4 (1.4)
Prohibit their sale on websites or online channels	118 (21.5)	80 (14.6)	350 (64.9)	3.8 (1.4)
No one can possess them	182 (33.2)	125 (22.8)	241 (44.0)	3.2 (1.5)

Around 28.8% of participants agreed, and 21.9% were undecided on whether e-cigarettes could help a person to quit smoking. Most disagreed that e-cigarettes were safer than traditional cigarettes (72.6%); thought that they could help them get along with friends (77.6%); indicated that they belonged to a new and trendy generation (79%); and that the image of an e-cigarette made them want to try one (74.5%).

The policy disagreement was 33.2% for the law that people could not have e-cigarettes in their possession, 29.9% for the import of e-cigarettes being strictly prohibited, 28% for a jail sentence of up to 10 years and/or a fine of five times the cost of the e-cigarettes for anyone who imports e-cigarettes, 25.9% for e-cigarettes being illegal, and 21.5% for selling e-cigarettes on websites or online channels being prohibited.

### Factors associated with e-cigarette use

Table 3 presents the participants’ characteristics associated with reporting ever using e-cigarettes. Males were more likely to report ever using e-cigarettes than females (29.0% vs 16.8%, p=0.001). Faculty groups were associated with reports of ever using e-cigarettes, with the highest report among International College (44.4%), Sciences and Technology (23.5%), Social and Arts (22.2%), and Health Sciences (17.0%) (p=0.001). The students with a monthly income of over 10000 THB were more likely to report ever using e-cigarettes (35.1% vs 17.1%, p<0.001). Having friends using e-cigarettes was associated with reports of using e-cigarettes (26.5% vs 2.8%, p<0.001).

**Table 3:** Characteristics of Thai university students associated with the use of e-cigarettes, 2022–2023 (N=548)

*Characteristics*	*Ever use e-cigarettes*		*p*
	*Yes n (%)*	*No n (%)*	
**Gender**			0.001**
Male	45 (29.0)	110 (71.0)	
Female	66 (16.8)	326 (83.2)	
Other	1 (100.0)	0 (0.0)	
**GPA**			0.130
<2.00	0 (0.0)	15 (100.0)	
2.01–3.00	27 (22.1)	95 (77.9)	
3.01–4.00	85 (20.7)	326 (79.3)	
**Faculties**			0.001**
Health Sciences	62 (17.0)	302 (83.0)	
Sciences and Technology	20 (23.5)	65 (76.5)	
Social and Arts	14 (22.2)	49 (77.8)	
International College	16 (44.4)	20 (55.6)	
**Family monthly income** (THB)			0.164
>50000	64 (22.8)	217 (77.2)	
≤50000	48 (18.0)	219 (82.0)	
**Personal monthly income** (THB)			<0.001***
>10000	40 (31.5)	87 (68.5)	
≤10000	72 (17.1)	349 (82.9)	
**Have friends who use e-cigarettes**			<0.001***
Yes	108 (26.5)	299 (73.5)	
No	4 (2.8)	137 (97.2)	

** Chi-squared test, significance at p<0.01.

*** Chi-squared test, significance at p<0.001.

Table 4 presents the bivariate relationships between attitudes toward e-cigarette use, perceived risk, policy opinions, age, and e-cigarette use. The mean attitude scores were significantly higher among those who reported ever using e-cigarettes than those who reported never using e-cigarettes (13.1 vs 8.9, p<0.001). The mean perceived risk scores were higher among those reported never using e-cigarettes than those reported ever using e-cigarettes (18.1 vs 15.9, p<0.001). In addition, the mean policy opinion agreement scores were higher among those who reported never using e-cigarettes than those ever using e-cigarettes (18.4 vs 12.3, p <0.001). In addition, when compared among those who ever used e-cigarettes (n=112), the mean of attitudes (14.2 vs 12.1, p<0.038) and the policy opinions (9.7 vs 14.3, p<0.020) were significantly different between those who used and did not use e-cigarettes last month. However, the mean age was not significantly different between those who reported ever using e-cigarettes and those who never used e-cigarettes.

**Table 4:** The association between attitudes, perceived risk, policy opinions, age, and e-cigarette use among Thai university students, 2022–2023

*Variables*	*Range*	*Ever use e-cigarettes (N=548) Mean (SD)*		*Used e-cigarettes last month (N=112) Mean (SD)*	
		*Yes (N=112)*	*No (N=436)*	*Yes (N=49)*	*No (N=63)*
**Attitudes^a^** (5 items)	5–25	13.1 (4.7)	8.9 (3.8)	14.2 (4.5)	12.1 (4.6)
p		<0.0 01***	0.0 38*		
**Perceived risks^b^** (4 items)	4–20	5.9 (3.7)	18.1 (2.8)	15.0 (3.7)	16.7 (3.6)
p		<0.0 01***	0. 051		
**Policy opinions^c^** (5 items)	5–25	12.3 (6.5)	18.4 (6.0)	9.7 (4.9)	14.3 (6.9)
p		<0.0 01***	0.0 20*		
**Age** (years)	18–26	20.5 (1.4)	20.5 (1.6)	20.1 (1.3)	20.8 (1.5)
p		0.945	0.520		

a Attitudes were measured as summative scores of 5 items (total score=25).

b Perceived risks were measured as summative scores of 4 items (total score=20).

c Policy opinions were measured as summative scores of 5 items (total score=25).

* Mann-Whitney U test, significance at p<0.05.

*** Mann-Whitney U test, significance at p<0.001.

Table 5 shows the logistic regression analysis. After controlling for other variables, attitudes, perceived risks, policy opinion, personal income, and friend use of e-cigarettes were significant predictors of reports ever using e-cigarettes. The likelihood of using e-cigarettes increased by 15% (AOR=1.15; 95% CI: 1.08–1.22) with each one-unit increase in attitude score. Conversely, the likelihood of using e-cigarettes decreased by 11% (AOR=0.89; 95% CI: 0.82–0.96) with each one-unit increase in perceived risk score. Additionally, the likelihood of using e-cigarettes decreased by 7% (AOR=0.93; 95% CI: 0.89–0.97) with each one-unit increase in policy opinion score.

**Table 5:** Logistic regression analysis to determine the relationship between the use of e-cigarettes among Thai university students and predictor variables (N=548)

	*Ever use e-cigarette ^a^*				*Used e-cigarettes during the last month ^b^*			
	*AOR^c^*	*95% CI*		*p*	*AOR^c^*	*95% CI*		*p*
		*Lower*	*Upper*			*Lower*	*Upper*	
Attitudes^d^	1.15	1.08	1.22	<0.001***	1.16	1.07	1.25	<0.001***
Perceived risks^d^	0.89	0.82	0.96	0.003**	0.88	0.80	0.98	0.015*
Policy opinions^d^	0.93	0.89	0.97	<0.001***	0.86	0.80	0.93	<0.001***
Gender (male)^e^	1.02	0.59	1.76	0.939	0.99	0.46	2.13	0.980
Age^d^	0.94	0.79	1.11	0.444	0.78	0.60	1.01	0.620
Personal income (>10000 THB)^e^	1.92	1.05	3.50	0.034*	1.17	0.49	2.76	0.723
Family income (>50000 THB)^e^	0.74	0.42	1.28	0.277	1.03	0.46	2.29	0.948
Friend use^e^	7.25	2.48	21.23	<0.001***	7.07	0.89	56.00	0.064
**Faculties^e^**								
Health Sciences (Ref.)				0.325				0.146
Science and Technology	1.06	0.53	2.11	0.875	2.68	1.07	6.69	0.035*
Social and Arts	1.41	0.64	3.14	0.395	1.65	0.53	5.16	0.386
International College	2.20	0.91	5.34	0.080	2.31	0.76	7.01	0.141

a Dependent variable was coded as 0=never use an e-cigarette, 1=use e-cigarette at least once.

b Dependent variable was coded as 0=did not use e-cigarette during last month, 1=used e-cigarette during last month.

c AOR: adjusted odds ratio.

d Predictor variables entered as continuous variables (attitudes, perceived risks, policy opinions, age).

e Predictor variables entered as categories (personal income, family income, friends use e-cigarettes, faculty).

* Statistically significant at p<0.05.

** Statistically significant at p<0.01.

*** Statistically significant at p<0.001.

Significant predictors for those reporting e-cigarette use last month included attitudes, perceived risks, policy opinions, and science and technology faculty enrollment. E-cigarette use last month increased by 16% (AOR=1.16; 95% CI: 1.07–1.15) with each attitude score increase. Conversely, it decreased by 12% (AOR=0.88; 95% CI: 0.80–0.98) with each perceived risk score increase, and by 7% (AOR=0.86; 95% CI: 0.80–0.93) with each policy opinion score increase.

## DISCUSSION

Although Thailand has laws and regulations prohibiting the use of e-cigarettes in the country, the results of our study indicate that about 20% of university students used e-cigarettes at least once, and almost 10% have used e-cigarettes during the last month. The results are similar to previous studies from universities in Vietnam^20^, where the prevalence of ever use of e-cigarette was 20.4%^20^. The highest proportion of e-cigarette users was from the International College group, followed by Sciences and Technology, then Social Sciences and Arts, and then Health Sciences. This observation could be attributed to the possibility that students from non-health-related faculties might be less aware of the risks associated with using e-cigarettes, which agrees with a previous study^21^. Nevertheless, it is worth noting that 70% of the study participants were female, which could potentially explain the lower proportion of e-cigarette users in our study. Previous research has indicated that males were more inclined to use e-cigarettes^27,28^.

This study identified perceived risk, attitudes, and policy opinions as significant predictors of e-cigarette use among university students. Furthermore, university students who had friends using e-cigarettes were more likely to use them than those with friends who did not. In addition, participants with a personal income exceeding 10000 THB per month demonstrated a higher likelihood of e-cigarette use compared to those with lower income. These findings were consistent with previous research conducted at a university in northern Thailand^21^.

Overall, more than 70% of students had negative attitudes toward e-cigarettes, which is similar to most previous research in this area^18,21,29^. However, around 50% agreed or were unsure that e-cigarettes could help them to quit smoking. Similarly, research with university students in Qatar found that around 70% of students agreed or were unsure that e-cigarettes prevented people from smoking conventional cigarettes^29^. However, around 51% of participants in our study support Thailand’s policies and laws that ban e-cigarettes, slightly higher than 47% of participants from research in Northern Thailand^21^.

The university students in our study had a higher perceived risk of e-cigarette use than participants in other research studies^18^. More than 80% to 90% of students agreed that e-cigarettes were addictive, could harm people’s lungs and cardiovascular system, could cause oral health problems, and that secondhand exposure to e-cigarette vapor was harmful. However, this contrasts with a study in Qatar, where only around 50% of students agreed with these topics^29^. Although the participants in the present study had a high perceived risk score, they still thought that e-cigarettes could help them quit conventional cigarette smoking. Hence, governments and universities must raise awareness among youth and college students regarding the nicotine dependence associated with e-cigarettes^30^. It is also crucial to emphasize that research findings remain uncertain whether e-cigarettes can effectively aid in smoking cessation^31^.

The participants in this study mentioned seeing e-cigarette advertising mainly through social media platforms, such as Instagram and Facebook, similar to previous research conducted in Singapore^32^. The university students in our study reported seeing e-cigarette media via TikTok more than YouTube, which is slightly different from the adult participants from a previous study in Singapore, which may be due to the popularity of TikTok over YouTube in the younger generation in Thailand. Additionally, a previous study indicated a significant association between online information exposure (including social media, websites, and total internet exposure) and the intention to use e-cigarettes^33^. This underscores the importance of enforcing regulations on online e-cigarette content.

Our research revealed that 40% of e-cigarette users were uncertain about the nicotine content in the e-cigarettes they consumed, indicating a lack of awareness regarding nicotine levels. Consequently, advocates for the younger generation must highlight the elevated nicotine levels in e-cigarettes, which can lead to increased nicotine dependence and pose long-term health risks. Additionally, e-cigarette use was associated with a higher likelihood of subsequently initiating cigarette smoking and engaging in cigarette use within the past 30 days^34^. Given these findings, addressing misconceptions and enhancing risk perceptions regarding e-cigarettes among university students, is imperative. To achieve this, mass media outlets should utilize diverse channels to reach at-risk groups within universities, including male students, those enrolled in non-health science programs, individuals from higher income backgrounds, and those with social circles and friends who use e-cigarettes.

### Limitations

This study has several limitations. Its cross-sectional design cannot establish causal relationships between e-cigarette use and interest variables such as attitudes, perceived risks, and policy opinions. Secondly, the findings may not be applicable or generalizable to other university student populations. Additionally, since e-cigarette use is illegal in Thailand, there is a possibility of response bias in the study. Lastly, the study was conducted among convenience samples of students who agreed to participate in the questionnaire, potentially leading to results that do not accurately represent the overall student population, which could affect the prevalence and characteristics of e-cigarette users.

## CONCLUSIONS

The study findings revealed significant associations between e-cigarette use and attitudes, perceived risk, and policy opinions among Thai university students. Students who used e-cigarettes tended to perceive lower risk, disagreed more with e-cigarette control policies, and exhibited positive attitudes towards e-cigarettes. Higher personal income and having friends who use e-cigarettes were also correlated with e-cigarette use. In addition, current e-cigarette users were more likely to study in science and technology faculties.

## Supplementary Material



## Data Availability

The data supporting this research are available from the authors on reasonable request.
