# Supplementary material for: E-cigarette use, perceived risks, attitudes, opinions of e-cigarette policies, and associated factors among Thai university students
Source: Tob Induc Dis. 2024 May 11;22:10.18332/tid/186536. doi: 10.18332/tid/186536 (PMC11087886; doi:10.18332/tid/186536)
Supplement: Supplementary file 1 [file TID-22-74-s1.pdf]

Figure 1. Reports of situations using e-cigarettes among Thai university students, 2022-2023 (can choose more than one answer) (N = 112)

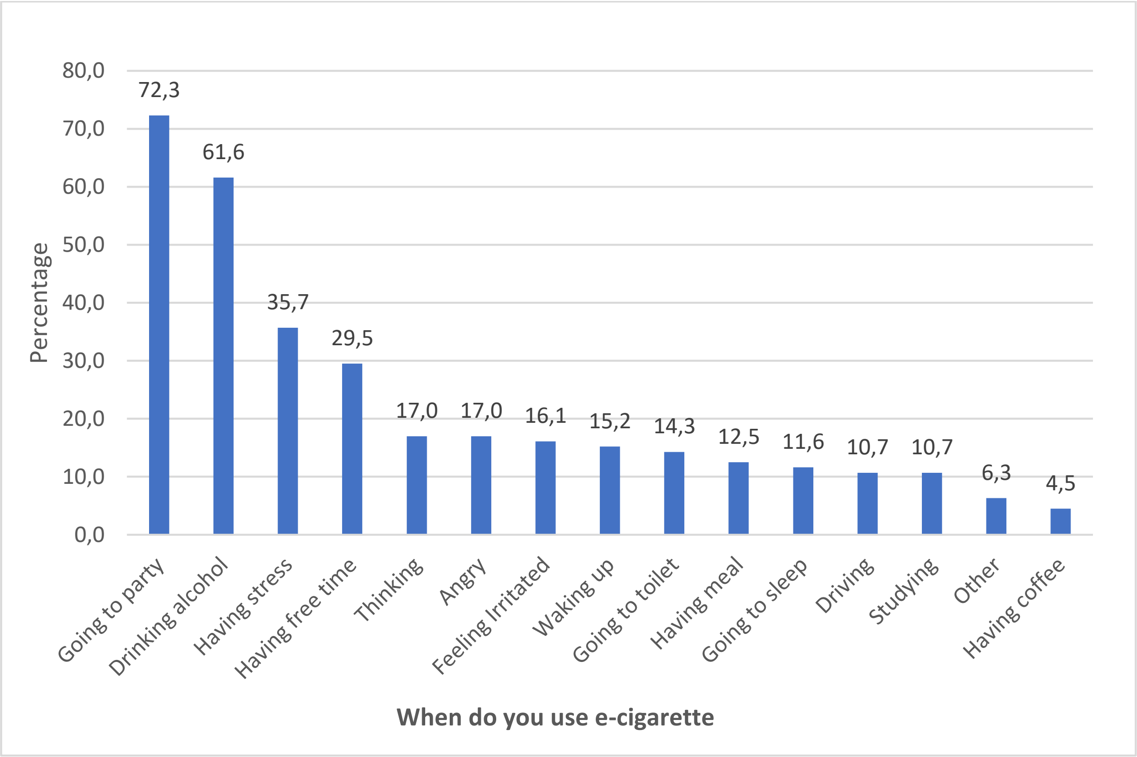

Figure 2. Main reason to start using e-cigarettes among Thai university students, 2022-2023 (N = 112)

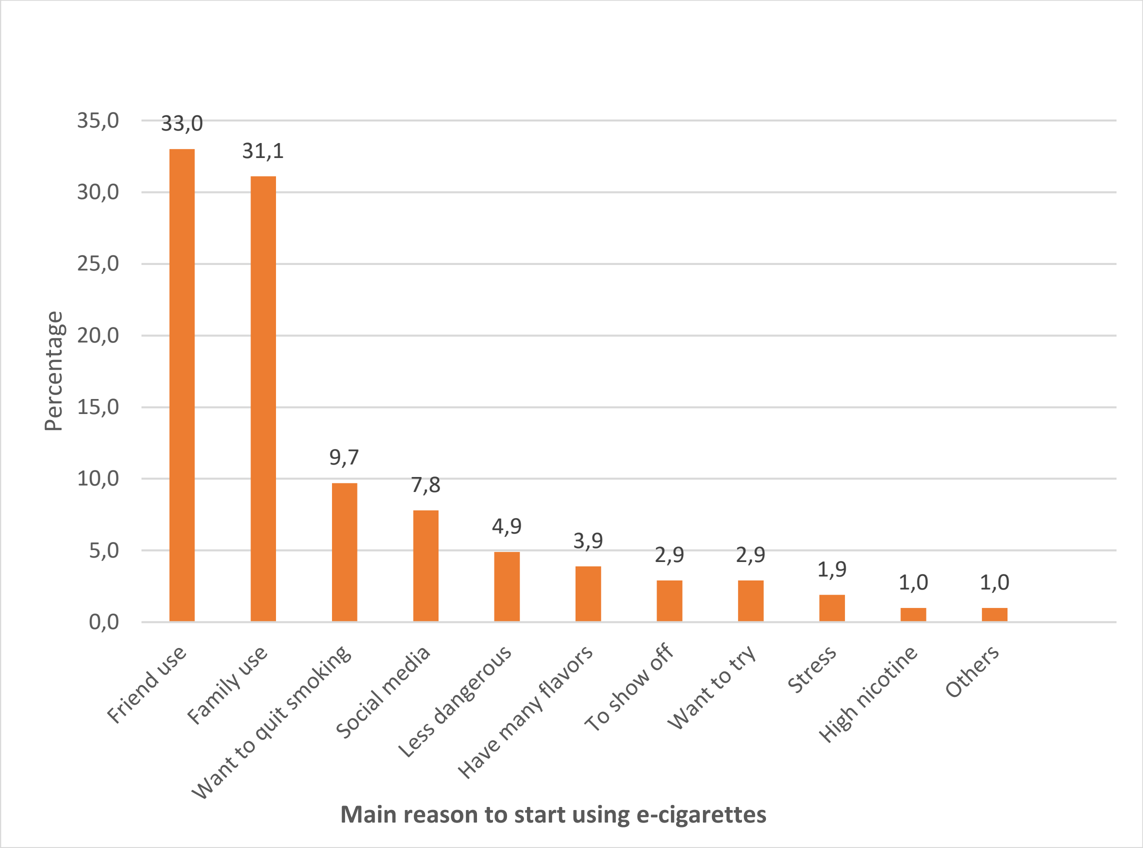

Figure 3. Frequency and types of media seen on e-cigarettes among Thai university students, 2022-2023 (N = 548)

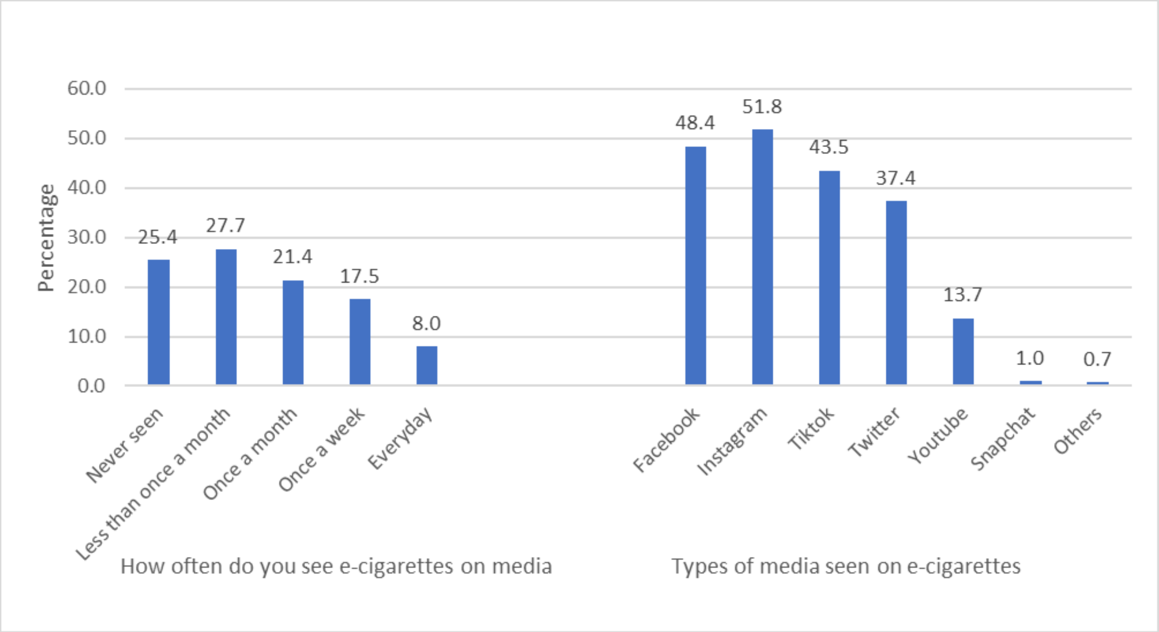

©2024 Vichayanrat T. et al.
